# Differences in recognition of similar medication names between pharmacists and nurses: a retrospective study

**DOI:** 10.1186/s40780-015-0017-4

**Published:** 2015-07-07

**Authors:** Toshikazu Tsuji, Toshihiro Irisa, Shinji Tagawa, Takehiro Kawashiri, Hiroaki Ikesue, Chiyo Kokubu, Akiko Kanaya, Nobuaki Egashira, Satohiro Masuda

**Affiliations:** Department of Pharmacy, Kyushu University Hospital, 3-1-1 Maidashi, Higashi-ku, Fukuoka, 812-8582 Japan

**Keywords:** Pharmacists, Nurses, Error rates, Preparation errors, Incidents, Similar medication names

## Abstract

**Background:**

Differences in error rates between pharmacists and nurses in terms of drug confirmation have not been studied. The purpose of this study was to analyze differences in error rates between pharmacists and nurses from the viewpoint of error categories, and to clarify differences in recognition regarding drug name similarity.

**Methods:**

In this study, preparation errors and incidents were classified into three categories (drug strength errors, drug name errors, and drug count errors) to investigate the influence of error categories on pharmacists and nurses. In addition, errors in two categories (drug strength errors and drug name errors) were reclassified into another two error groups, to investigate the influence of drug name similarity on pharmacists and nurses: a “drug name similarity (−) group” and a “drug name similarity (+) group”. Then, differences in error rates of pharmacists and those of nurses were analyzed respectively within three categories and two groups. Furthermore, differences in error rates between pharmacists and nurses were analyzed in each of the three categories and two groups.

**Results:**

Error rates of pharmacists for both drug strength errors and drug name errors were significantly higher than that for drug count errors, and similar results were obtained for nurses (*P *< 0.05). However, there were no significant differences in error rates between pharmacists and nurses in each of the three categories. Furthermore, error rate of nurses was significantly higher than that of pharmacists in the drug name similarity (+) group (*P *< 0.05), while there was no significant difference in error rates between pharmacists and nurses in the drug name similarity (−) group.

**Conclusions:**

These results suggest that in contrast to pharmacists, nurses are easily affected by similarities in drug names. Therefore, pharmacists should offer information on medications having plural strengths or similar names to nurses, in order to minimize damage to patients resulting from errors.

**Electronic supplementary material:**

The online version of this article (doi:10.1186/s40780-015-0017-4) contains supplementary material, which is available to authorized users.

## Background

Though standards of security in healthcare have advanced, medical incidents and accidents continue to occur. In the department of pharmacy at Kyushu University Hospital, we have been working on countermeasures to prevent incidents regarding oral and external medications. We have maintained an occurrence rate of these incident types in the range of 0.027–0.036% for eight years, since April 2006 [1-4]. As a matter of course, pharmacists are fully accountable for these incidents. Therefore, pharmacists should make every effort to prevent incidents caused by their own errors. On the other hand, it is also important for pharmacists to recognize the categories of drug errors that are liable to be overlooked by pharmacists, lead to administration to patients, and cause serious damage to patients. On a practical level, it is impossible for pharmacists to prevent administration after the delivery of incorrect medications. Concerning the management of inpatient medication, nurses check all medications prior to administration in our hospital. Therefore, it is clear that nurses play an important role in preventing administration of incorrect drugs. In short, nurses minimize the patient damage by detecting the mistakes overlooked by pharmacists.

Many reports exist regarding prevention measures for incidents caused by pharmacists [1,8], and many analytical studies have been conducted regarding the probability of drug name confusion [9–14]. However, differences in medication error rates between pharmacists and nurses have not yet been studied.

In the present study, preparation errors and incidents were classified into three categories (drug strength errors, drug name errors, and drug count errors). Furthermore, drug strength and drug name errors were classified into two groups: drug name similarity (−) or drug name similarity (+). Then, differences in error rates between pharmacists and nurses were analyzed within and across these three categories and two groups.

## Methods

### Study period and subject of investigation

The study period lasted eight years, from April 2006 to March 2014. Preparation errors and incidents regarding oral medications among inpatient prescriptions were investigated. Among these, errors pertaining to narcotics, powders, and tablets divided by the automatic packaging machine were excluded from investigation, because of the difference in dispensing procedures. Furthermore, the investigation was restricted to errors that could be classified into three categories (drug strength, drug name, and drug count errors).

Preparation error data was self-reported by pharmacy inspectors, and incident data was reported by nurses. Also, it was not necessary to obtain written informed consent by each patient in the present retrospective study based on the ethical guidelines for clinical studies by Ministry of Health, Labor and Welfare, Japan. The individual information concerning patients was protected appropriately. In addition, problems regarding the occurrence of preparation errors by pharmacists were not a concern, because these errors were regarded as the population parameter for the calculation of error rates of pharmacists in this study.

### Definition of incidents and classification of incident impact on patients

We defined errors detected by nurses or inpatients after being overlooked by pharmacy inspectors as “incidents”. According to the provisions of the National University Hospital in Japan, the impact on patients of the incidents was classified into six stages (Levels 0–5) as described below.

Level 0: Incorrect drug was delivered to the nurse, but it was not taken by a patient.

Level 1: Incorrect drug was taken by a patient, but patient was not adversely affected.

Level 2: Moderate impact to the patient, but treatment was not needed.

Level 3: Provisional or continual treatment was needed.

Level 4: Severe impact on the patient remained.

Level 5: Patient died.

### Definition of preparation errors, incidents more than Level 0, and incidents more than Level 1

We defined errors detected by pharmacy inspectors as “preparation errors”, errors not detected by pharmacy inspectors as “incidents more than Level 0”, and errors that led to administration after being overlooked by nurses as “incidents more than Level 1”. In this study, practical preparation errors were considered equivalent to an “all errors” that including incidents more than Level 0, because these incidents were simply not detected by the pharmacists at the point of inspection.

In short, the number of preparation errors included that of incidents more than Level 0, and the number of incidents more than Level 0 included that of incidents more than Level 1. The definition of preparation errors, incidents more than Level 0, and incidents more than Level 1 was summarized as described below.

Preparation errors: Errors that were revealed to be incorrect afterward. These were equivalent to an “all errors” category, and included errors detected by pharmacy inspectors.

Incidents more than Level 0: Errors that were not detected by pharmacy inspectors and led to delivery of medication to nurses.

Incidents more than Level 1: Errors that were not detected by nurses and led to administration of medication to patients.

### Classification of preparation errors and incidents into three categories

Preparation errors and incidents were classified into three categories (drug strength, drug name, and drug count errors) to investigate the influence of error categories on pharmacists and nurses. The error rates of pharmacists were calculated by dividing the number of incidents more than Level 0 by that of preparation errors (incidents more than Level 0/preparation errors). The error rates of nurses were calculated by dividing the number of incidents more than Level 1 by that of incidents more than Level 0 (incidents more than Level 1/incidents more than Level 0). Then, differences in error rates of pharmacists and those of nurses were analyzed respectively within three categories. Furthermore, the differences in the error rates between pharmacists and nurses were analyzed in each of three categories.

### Reclassification of preparation errors and incidents into two groups

Trade names of Japanese drugs are expressed by *katakana* in most cases. In Japanese, *katakana* expressions consists of both orthographic (i.e., spelling) and phonological (i.e., pronunciation) aspects. In the present study, *katakana* trade names were converted into Romanized versions of Japanese (non-English words Romanized using Hepburn's method), to represent the exact features of the *katakana*.

In order to investigate the influence of drug name similarity on pharmacists and nurses, preparation errors and incidents in two categories (drug strength errors and drug name errors) were first totaled. Then, these errors were reclassified into two further error groups: “having less than four letters in common” or “having more than five letters in common”, from the viewpoint of drug name similarity.

If the correct drug and incorrect drug had the same drug strength, we defined this as equivalent to “one additional letter” in terms of having continuous letters in common. For example, “*PU/RA*/BI/KKU/SU (*75*)” and “*PU/RA*/ZA/KI/SA (*75*)” share four letters and drug strength; the underlines represent the common points between them. In this case, we defined this error group as “having more than five letters in common”. Furthermore, we defined the error group “having less than four letters in common” as the “drug name similarity (−) group”, and the error group “having more than five letters in common” as the “drug name similarity (+) group”. Additional file 1: Table S1 shows the classification of the errors into two groups and examples of errors.

Then, the differences in error rates of pharmacists and those of nurses were analyzed respectively within the two groups. Furthermore, the differences in the error rates between pharmacists and nurses were analyzed in each of two groups.

### Data analysis

Data were analyzed with a chi-square test. *P* values of <0.05 were considered statistically significant, and *P* values of <0.1 were considered marginally statistically significant. Differences in error rates of pharmacists and those of nurses were analyzed respectively among three categories and between two groups. In addition, the differences in the error rates between pharmacists and nurses were analyzed in each of three categories and two groups.

## Results

### Number of preparation errors, and incidents more than Level 0, 1, and 2

Over the eight years, 758,310 inpatient prescriptions were given. The number of preparation errors in the three categories (drug strength, drug name, and drug count errors) were 392,650, and 2,588, the number of incidents more than Level 0 were 38,73, and 117, the number of incidents more than Level 1 were 6, 9, and 2, and the number of incidents more than Level 2 were 2, 5, and 0, respectively. There were no incidents more than Level 3.

**Fig. 1:**
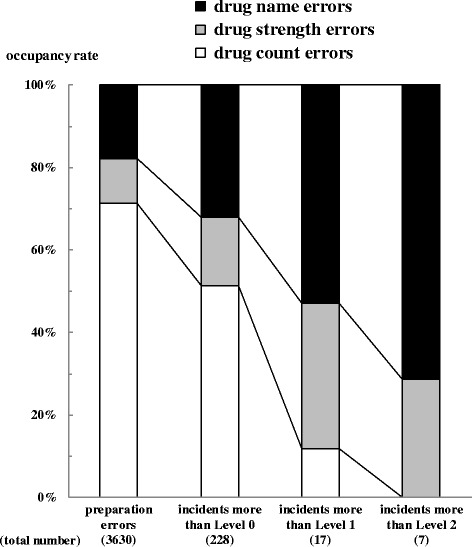
Occupancy rates of preparation errors, and incidents more than Level 0, 1, and 2 in three categories. The number in parenthesis indicates the total number of errors of three categories (drug count errors, drug strength errors, drug name errors). Occupancy rate indicates the percentage of the number of each category to the total number. The occupancy rates at the respective stages from “preparation errors” to “incidents more than Level 2” are indicated according to three categories

**Fig. 2:**
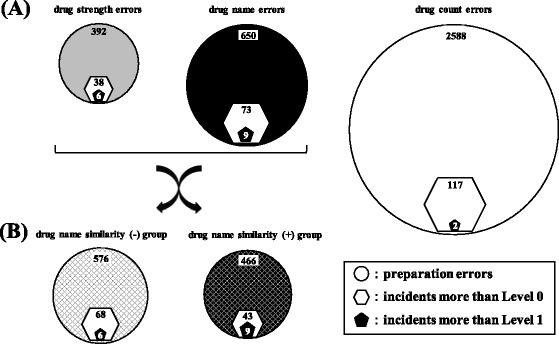
Schematic view of the number of preparation errors, incidents more than Level 0, and incidents more than Level 1 in three categories **(a)** and two groups **(b)**. Preparation errors and incidents were classified into three categories **(c**: drug strength errors, drug name errors, drug count errors). And these errors in two categories (drug strength errors, drug name errors) were reclassified into another two groups **(b**: drug name similarity (−) group, drug name similarity (+) group). Circles indicate the number of preparation errors, hexagons indicate the number of incidents more than Level 0, and pentagons indicate the number of incidents more than Level 1, respectively. The number of preparation errors includes that of incidents more than Level 0, and the number of incidents more than Level 0 includes that of incidents more than Level 1

Figure 1 shows the occupancy rates for each stage from “preparation errors” to “incidents more than Level 2” in the three categories. The occupancy rates of preparation errors, incidents more than Level 0, 1, and 2 in the category of drug count errors were 71.3% (2588/3630), 51.3% (117/228), 11.8% (2/17), and 0% (0/7), respectively. In contrast, the same rates in the category of drug name errors were 17.9% (650/3630), 32.0% (73/228), 52.9% (9/17), and 71.4% (5/7), respectively. In addition, the same rates in the category of drug strength errors were 10.8% (392/3630), 16.7% (38/228), 35.3% (6/17), and 28.6% (2/7), respectively. Furthermore, Fig. 2 shows the schematic view of the number of preparation errors, incidents more than Level 0, and incidents more than Level 1 in three categories (a) and two groups (b).

### Error rates of pharmacists and nurses in three categories

**Fig. 3:**
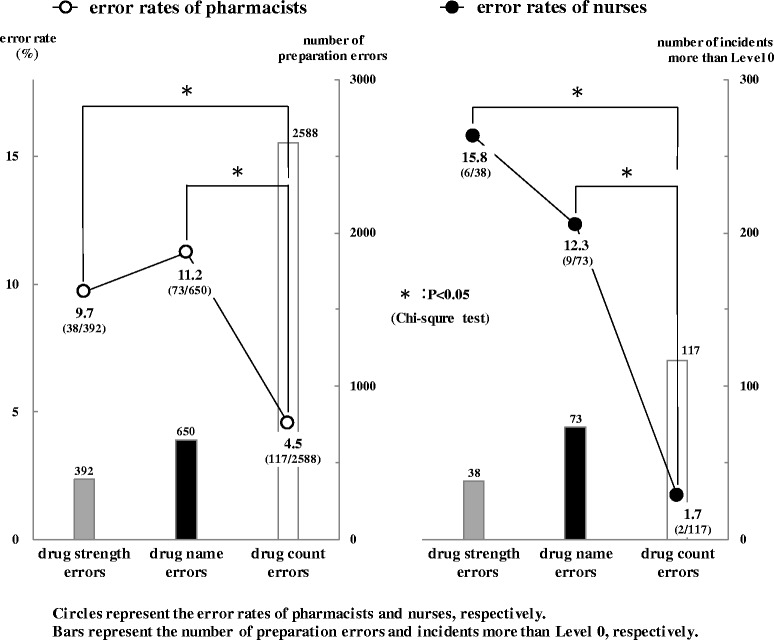
Error rates of pharmacists and nurses in three categories. Preparation errors and incidents were classified into three categories (drug strength errors, drug name errors, drug count errors). Open circles indicate the error rates of pharmacists and closed circles indicate the error rates of nurses, respectively. Bars in the left figure indicate the number of preparation errors and bars in the right figure indicate the number of incidents more than Level 0, respectively. Data were analyzed with a chi-square test. *P* values of <0.05 were considered statistically significant

Figure 3 shows the error rates of pharmacists and nurses in three categories. Distributions of both preparation errors and incidents more than Level 0 showed a similar tendency in the following order: drug strength errors < drug name errors < drug count errors.

Error rates of pharmacists in three categories (drug strength, drug name, and drug count errors) were 9.7% (38/392), 11.2% (73/650), and 4.5% (117/2588), respectively. In addition, the error rate of pharmacists in the category of drug name errors was the highest among the three categories. The respective error rates of pharmacists in the categories of both drug strength errors and drug name errors were significantly higher than for the category of drug count errors (*P* < 0.05).

In contrast, the error rates of nurses in the three categories (drug strength, drug name, and drug count errors) were 15.8% (6/38), 12.3% (9/73), and 1.7% (2/117), respectively. Error rate of nurses in the category of drug strength errors was the highest among the three categories. Furthermore, the respective error rates of nurses in the categories of both drug strength errors and drug name errors were significantly higher than for the category of drug count errors (*P* < 0.05). In short, these results suggest that nurses are good at detecting drug count errors, but poor at detecting drug strength errors.

Furthermore, there were no significant differences in error rates between pharmacists and nurses in each of the three categories. Among them, difference in error rates between pharmacists and nurses was greatest in the category of drug strength errors.

### Error rates of pharmacists and nurses in two groups

**Fig. 4:**
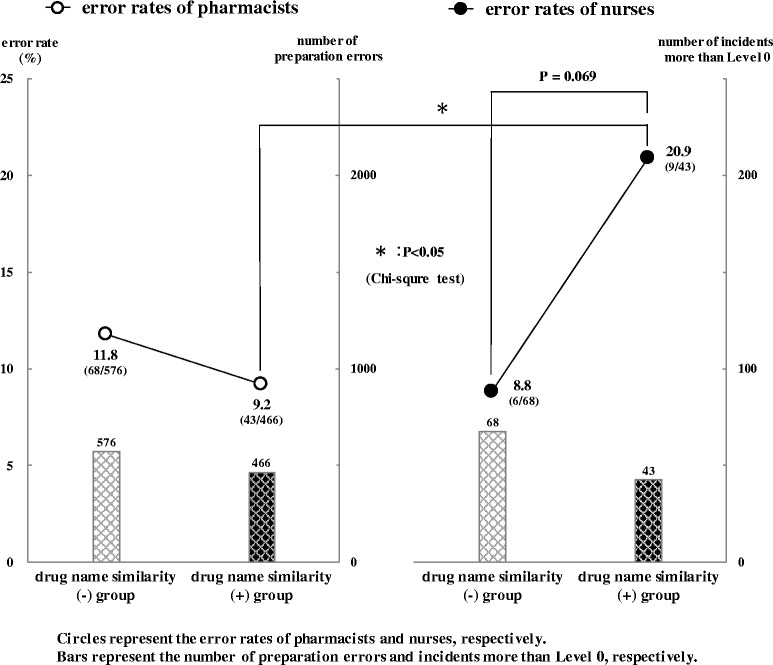
Error rates of pharmacists and nurses in two groups. Preparation errors and incidents in two categories (drug strength errors, drug name errors) were reclassified into another two groups (drug name similarity (−) group, drug name similarity (+) group). Open circles indicate the error rates of pharmacists and closed circles indicate the error rates of nurses, respectively. Bars in the left figure indicate the number of preparation errors and bars in the right figure indicate the number of incidents more than Level 0, respectively. Data were analyzed with a chi-square test. *P* values of <0.05 were considered statistically significant, and *P* values of <0.1 were considered marginally statistically significant

Figure 4 shows the error rates of pharmacists and nurses in two groups. The distributions of both preparation errors and incidents more than Level 0 showed a similar tendency: drug name similarity (−) group > drug name similarity (+) group.

The respective error rates of pharmacists in the drug name similarity (−) and (+) group were 11.8% (68/576) and 9.2% (43/466), and there was no significant difference in the error rates of pharmacists between the two groups. On the other hand, the respective error rates of nurses in the drug name similarity (−) and (+) group were 8.8% (6/68) and 20.9% (9/43), and there was a marginally significant difference in the error rates of nurses between the two groups (*P* = 0.069).

Furthermore, there was no significant difference in error rates between pharmacists and nurses (11.8% and 8.8%) in the drug name similarity (−) group. On the other hand, there was a significant difference in error rates between pharmacists and nurses (9.2% and 20.9%) in the drug name similarity (+) group (*P* < 0.05). In short, these results suggest that nurses are easily affected by similarities in drug names in contrast to pharmacists.

## Discussion

The occupancy rates of preparations errors, incidents more than Level 0, 1, and 2 in the category of drug count errors decreased gradually in accordance with a rise in impact to the patient. In contrast, the same rates in the category of drug name errors increased gradually, and a similar tendency was seen in the category of drug strength errors. In short, greater impact on patients is seen in the following order: drug name errors > drug strength errors > drug count errors. Furthermore, the transition rate from “preparation errors” to “incidents more than Level 0” was highest for drug name errors (11.2%; 73/650). In addition, the same rate from “incidents more than Level 0” to “incidents more than Level 1” was highest for drug strength errors (15.8%; 6/38). Finally, the same rate from “incidents more than Level 1” to “incidents more than Level 2” was highest for drug name errors (55.6%; 5/9). These results suggest that pharmacists tend to make drug name errors, nurses tend to make drug strength errors, and inpatients tend to suffer serious damage after taking incorrect drugs, which is related to drug name errors.

Again, pharmacists are fully accountable for these incidents, because the root causes of them are made by the pharmacists. Therefore, in the first place, pharmacists should make every effort to keep the incidents to the minimum. In addition, pharmacy inspectors should prevent preferentially the high-risk incidents by recognizing that expansion of patient damage is caused in the following order: drug name errors > drug strength errors > drug count errors. As a countermeasure for preventing these mistakes, pharmacy inspectors are working on confirming thoroughly “identification code” indicated on the exterior of each medication. Medication identification codes are indicated on the prescription through coordination at our hospital pharmacy. Therefore, it is possible to compare medication and prescription codes. For example, the identification codes: “*PURA*BIKKUSU (*75*)”, “*PURA*ZAKISA (*75*)”, “*PURA*BIKKUSU (25)”, and “*PURA*ZAKISA (110)” are expressed as “sa 75”, “R 75”, “sa 25”, and “R 110” respectively (The underlines represent the common points among trade names in Romanized Japanese). Because the identification code is typically a simple and unique combination of numbers, symbols, and so on, it is unlikely for pharmacy inspectors to be influenced by preconceptions in terms of comparing the two codes. In fact, the error rate of pharmacists in the category of drug count errors was significantly lower than that of the other two categories (drug strength and drug name errors), and the same results were obtained for the error rates of nurses. These results suggest that confirmation utilizing numerical values or symbols would be a simple and effective method that would not be affected by preconceptions.

From the viewpoint of drug name similarity, error rate of nurses was significantly higher than that of pharmacists in the drug name similarity (+) group (20.9%, 9.2%; *P* < 0.05). Furthermore, error rate of nurses in the drug name similarity (+) group tended to be higher than that in the drug name similarity (−) group (20.9%, 8.8%; *P* = 0.069). In other words, compared to pharmacists, nurses are easily affected by similarities in drug names. These results suggest that there is a difference in recognition regarding similarities in drug names between pharmacists and nurses. The main reason for these errors by nurses is likely to be a lack of knowledge of the medications that cause the risk of name confusion.

However, unlike pharmacists, nurses cannot confirm identification codes in hospital wards or at nurse stations. Therefore, it is necessary for pharmacists to offer information on medications having multiple strengths or similar names to nurses. For example, publishing a list of these medications would help nurses to recognize the presence of medications causing a risk for name confusion. Such measures would help in education on medical safety for nurses as well as pharmacists, and would lead to a subsequent reduction of serious damage to patients.

## Conclusions

Our results suggest that increasing damage is caused to patients by errors in the following order: drug name errors > drug strength errors > drug count errors. Therefore, pharmacists should make efforts specifically to prevent high-risk errors, such as drug name errors. Furthermore, there was no difference in error rates between pharmacists and nurses from the viewpoint of error categories, while there was a difference in error rates between them for drug name similarities. In short, in contrast to pharmacists, nurses are easily affected by similarities in drug names, which suggests a difference in recognition of drug names between pharmacists and nurses. Therefore, it is necessary for pharmacists to offer information to nurses on medications having multiple strengths or similar names, in order to minimize damage to patients due to medication errors.

## Additional file

Additional file 1: Table S1. Classification of errors into two groups and examples of errors. (PDF 169 KB)
