# Supplementary material for: Differences in recognition of similar medication names between pharmacists and nurses: a retrospective study
Source: J Pharm Health Care Sci. 2015 Jul 7;1:19. doi: 10.1186/s40780-015-0017-4 (PMC4728788; doi:10.1186/s40780-015-0017-4)
Supplement: Additional file 1: Table S1. — Classification of errors into two groups and examples of errors. (PDF 169 KB) [file 40780_2015_17_MOESM1_ESM.pdf]

Table S1. Classification of errors into two groups and examples of errors

The classification method of errors based on drug name similarity was defined using the following five steps

1. The similarity of drug names was compared by converting the *katakana* trade name into Romanized Japanese (Hepburn system).
2. According to the commonality of continuous letters in Romanized Japanese, errors were divided into two error groups: "having less than four letters in common" or "having more than five letters in common."
3. "A," "I," "U," "E," "O," and "N," which are pronounced independently, were defined as "two letters." Furthermore, "SHI," "CHI," and "TSU" were defined as "two letters."
4. If two medications had the same strength, this was equivalent to "one additional letter" in terms of common continuous letters.
5. The error groups, "having less than four letters in common" and "having more than five letters in common" were defined as "drug name similarity (-) group" and "drug name similarity (+) group," respectively.

"drug name similarity (-) group"

(correct drug name)		(incorrect drug name)	commonality of letters
MA/I/SU/RI/I (5)		RE/N/DO/RU/MI/N (0.25)	
I/SU/KO/CHI/N (100)	⇒	I/TO/RI/ZO/O/RU (50)	less than 2 letters
DE/KA/DO/RO/N (0.5)		DE/PA/SU (1)	
DE/KA/DO/RO/N (0.5)		DE/PA/SU (0.5)	
KA/RU/TA/N (500)	⇒	KA/RO/NA/A/RU (200)	3 letters
PU/RI/N/PE/RA/N (5)		PU/RU/ZE/NI/DO (12)	
WA/A/FUA/RI/N (1)		BA/I/A/SU/PI/RI/N (100)	
GU/RA/SE/PU/TA/A (1)	⇒	PU/RO/GU/RA/FU (0.5)	4 letters
PU/RA/BI/KKU/SU (25)		PU/RA/ZA/KI/SA (110)	

"drug name similarity (+) group"

(correct drug name)		(incorrect drug name)	commonality of letters
PU/RA/BI/KKU/SU (75)	⇒	PU/RA/ZA/KI/SA (75)	
PU/RO/SE/KI/SO/O/RU (0.5)		PU/RO/SU/TA/A/RU (25)	5 letters
TO/RA/MA/A/RU (25)		TO/RA/MU/SE/TTO (-)	
NO/I/RO/BI/TA/N (-)		NO/I/RO/TO/RO/PI/N (4)	
NO/RU/BA/DE/KKU/SU (20)	⇒	NO/RU/BA/SU/KU (5)	6 letters
RI/RI/KA (25)		RI/RI/KA (75)	
A/RU/FUA/RO/O/RU (1)		WA/N/A/RU/FUA (0.5)	
GA/BA/PE/N (300)	⇒	GA/BA/PE/N (400)	more than 7 letters
A/KU/TO/NE/RU (17.5)		A/KU/TO/NE/RU (75)	

The underlines represent the common points between correct drug and incorrect drug.
